# Post-TB treatment completion experiences of children, adolescents, and caregivers from Cape Town

**DOI:** 10.5588/ijtldopen.25.0117

**Published:** 2025-11-12

**Authors:** C. Monin, D.T. Wademan, C. Purdy, M. Mlomzale, M.G. Anthony, T. Cousins, L. Viljoen, J. Orne-Gliemann, M.M. van der Zalm, G. Hoddinott

**Affiliations:** 1 Desmond Tutu TB Centre, Department of Paediatrics and Child Health, Stellenbosch University, Cape Town, South Africa;; 2 National Institute for Health and Medical Research (INSERM), UMR 1219, Research Institute for Sustainable Development (IRD), EMR 271, Bordeaux Population Health Centre, University of Bordeaux, Bordeaux, France;; 3 Oxford School of Anthropology and Museum Ethnography, University of Oxford, Oxford, UK;; 4 Department of Sociology and Social Anthropology, Stellenbosch University, Cape Town, South Africa;; 5 School of Public Health, Faculty of Medicine and Health, The University of Sydney, Sydney, NSW, Australia.

**Keywords:** tuberculosis, Cape Town, South Africa, PTLD, health-related quality of life, HRQoL

## Abstract

**BACKGROUND:**

Post-TB life is associated with a range of clinical, economic, social, and psychological sequelae, with limited data available on children and adolescents. We describe child TB survivors’ physical, emotional, and social post-TB treatment experiences, in a high-incidence setting in South Africa.

**METHODS:**

An explorative qualitative study was nested within the Umoya TB cohort between June and September 2023. We used semi-structured interviews and participatory methods, including body mapping, to explore participants’ physical, emotional, and social wellbeing. Data were analysed using a deductive thematic approach and a health-related quality-of-life framework.

**RESULTS:**

Thirty semi-structured interviews were conducted with 15 children/adolescents; median age 9 years (range: 5–15); 8 (53%) were male; 2 (13%) living with HIV, and 1 (6%) had multidrug-resistant TB. Most interviews were conducted with children together with their caregivers (N = 14). Interviews were done 11–61 months (41-month average) after TB treatment completion. All participants reported that TB significantly impacted their physical, psychological, and social domains, extending well beyond treatment completion. Children and adolescents perceived changes in their bodies like shortness of breath and physical pain following their TB episode, reporting various physical post-TB cure symptoms. TB-related stigma disrupted participants’ social relationships, especially among adolescents. Broader underlying socio-environmental challenges exacerbated the long-term economic impact of TB on household financial instability.

**CONCLUSION:**

The negative impacts of TB extend well beyond children and adolescents’ treatment completion across multiple aspects of their lives. Future studies should prioritise the development of interventions to enhance communication and optimise follow-up care for paediatric TB survivors.

In 2020, an estimated 155 million individuals were TB survivors, many of whom remain at risk for post-TB morbidity. Among them, more than 18.6 million were children and adolescents (0–19 years old).^1^ Historically, national TB programmes have focused on the TB treatment phase, aiming to reduce TB disease incidence and improve diagnosis and treatment outcomes, with giving limited attention to the post-treatment period.^2^ However, there is increased awareness that despite successful TB treatment, TB survivors are at increased risk of all-cause mortality and long-term morbidity.^3^ This post-acute phase, with evidence of chronic respiratory abnormality, with or without symptoms, and attributable at least in part to previous pulmonary TB (PTB), has been described as post-TB lung disease (PTLD).^4^ Meta-analyses have shown that respiratory morbidity is present in >50% of TB survivors, who subsequently may experience reduced health-related quality of life (HRQoL).^5^ Even in the absence of lung impairment, TB survivors often experience a range of long-term economic, social, and psychological sequelae.^6^ Although PTLD is an emerging area of research, the majority of existing data come from adult survivors. The impact on children’s physical health has gained recent attention with children and adolescent TB survivors showing enduring respiratory symptoms, radiological sequelae, and impaired lung function.^7–10^ A prospective study in Cape Town in adolescents with microbiologically confirmed PTB during and after treatment completion found that 65% of adolescents with TB had abnormal lung function, compared with 58% of adolescents after treatment completion (odds ratio: 1.3, 95% confidence interval: 0.5, 3.3).^9^ The long-term impact of TB in children and adolescents, particularly respiratory impairment and disability, remains poorly understood due to limited research, inconsistent terminology, and a lack of suitable metrics for evaluation, hindering progress in this crucial area.^11–15^ A recent World Health Organization–commissioned review highlighted the fact that TB has multiple negative impacts on children and adolescents, which persist well beyond their successful treatment outcomes.^16^ These impacts are shaped by how TB care is delivered, such as through extended isolation or inpatient treatment, which often disrupts daily life, schooling, and family dynamics.^17^ A clinical statement on post-TB health and wellbeing reported that no TB-specific HRQoL measure exists and called for qualitative research into the socio-economic and mental health impact of post-TB among children.^18^ Data on children and adolescents’ experiences of TB disease suggest multiple economic, social, and psychological impacts on their HRQoL.^19–21^

We need to better understand the long-term impacts of TB on children and adolescents, particularly how they and their caregivers perceive changes to their physical, psychological, and social wellbeing following treatment completion. We explored children and adolescents’ lived experiences of how TB has enduring impacts on daily life, health, and outlook on the future even after treatment completion.

## METHODS

The Western Cape Province of South Africa has one of the highest burdens of paediatric TB in the world, with in 2022/2023 an estimated TB incidence of 681 per 100,000 population and 13% of notified TB cases being children.^22,23^ The City of Cape Town is an urban/peri-urban setting home to 4,756,255 people – ∼67% of residents in the province. The city delivers health services through 94 primary health care facilities, 4 district hospitals, and 2 tertiary hospitals.^23^ The population mainly consists of English-, Afrikaans-, and isiXhosa-speaking inhabitants with a large proportion living in poverty, overcrowded conditions, and informal housing. The Children and Adolescents Post-TB Experiences (CAPEs) study was nested within the Umoya study, an ongoing prospective observational TB diagnostic and lung health study cohort in Cape Town, South Africa.^24^ Briefly, children aged 0–13 years with presumptive PTB were recruited from Tygerberg Hospital and Karl Bremer Hospital, two regional referral centres, with an estimated paediatric TB caseload of ∼600 per year. The broader cohort includes children with TB and healthy controls and follows participants for up to 4 years post-enrolment. Children were followed up regularly to assess lung health outcomes and HRQoL.

### Study design, sampling, and recruitment

This is an exploratory qualitative study. Researchers recruited children and their caregivers from the Umoya study after they had completed TB treatment. Eligible participants were identified from the Umoya cohort based on age (5–17 years), prior TB diagnosis, and treatment completion. Purposive sampling was used to ensure diversity in age, sex, and language (Afrikaans, English, *isiXhosa*). A total of 83 eligible children were identified. The study team, in collaboration with Umoya recruiters, contacted caregivers and arranged interviews. Recruitment continued until theoretical saturation was reached, with a final sample size of 16 guided by similarly designed qualitative studies.^25^ The sample size does not allow for detailed exploration within groups (e.g., adolescents vs. younger children, or among participants living with HIV).

### Data collection

The semi-structured interview guide included topics to explore children’s experiences of TB disease and treatment, and the impact of TB beyond treatment completion on their lives. Interviews included open-ended questions on physical recovery, emotional wellbeing, stigma, social reintegration, and family dynamics post-treatment, as outlined in the discussion guide (see Supplementary Data). We engaged children and their caregivers in participatory research activities.^26^ Two of the interviewers were native speakers of isiXhosa and Afrikaans, respectively, and the third was fluent in English. Wherever possible, we encouraged children (regardless of their age) to report their experiences directly; alternatively, caregivers responded on behalf of their children. For younger children, interviews were adapted to their developmental stage, using simplified language and visual methods to encourage participation (body mapping). Interviewers had prior experience working with children in TB care and worked within the cultural context of the participants, contributing to their understanding of participants’ experiences. Caregivers were encouraged to contribute especially when children had limited memory of their TB experience. All interviews were conducted in the participants’ preferred language (Afrikaans, English, or *isiXhosa*). Interviews ranged from 30 to 90 min in duration and were audio-recorded and written as case descriptions. Participants were informed they could pause or stop the interview at any time and were informed of available psychological support if needed. We interviewed each participant (child/adolescent plus their caregiver[s]) twice, approximately 4 weeks apart. The first interview focused on TB diagnosis and treatment experiences, while the second explored post-TB challenges and longer-term impacts on health and wellbeing. Interviewers (graduate socio-behavioural scientists: CM, CP, and MM) kept detailed observational and reflexive notes, as well as copies of written and drawn activities completed by the participants. For example, the body mapping activity used a pre-drawn body outline and asked children to indicate areas affected during or after TB, using symbols or colours to represent physical and emotional experiences. These activities were used flexibly depending on the child’s age and abilities and were supported with open-ended prompts (e.g., ‘What part of your body felt different?’ or ‘Can you show us how TB made you feel?’).

### Data analysis

Interviewers compiled structured case descriptions, detailing participants’ experiences with TB, after each interaction with participants. We combined the two case descriptions from each participant into a single case file. Case files (n = 15) included field notes, and direct quotes drawn from audio recordings were shared and reviewed by a senior social scientist (DTW). Case descriptions were based on full audio recordings and included verbatim quotes. Case files were reviewed by multiple co-authors to ensure consistency of interpretation. A deductive thematic analysis was then conducted, using an adapted version of the HRQoL in PTB framework proposed by Kastien-Hilka and colleagues to drive the analysis of case files.^27,28^ Themes were identified using a structured coding approach guided by the HRQoL framework, and codes were iteratively refined through group discussions. Interpretation of findings was iteratively discussed among co-authors (CM, CP, MM, DTW, and TC) to ensure that all potential biases were identified and addressed throughout the process. This iterative approach helped ensure that the findings were fully elucidated, valid, and reflective of the participants’ experiences.

### Ethical statement

The Umoya study has ethics approval from Stellenbosch University Health Research Ethics Committee (HREC N17/08/083). The CAPEs study received additional ethics approval from the HREC at Stellenbosch University (N23/02/008_Sub Study N17/08/083). All caregivers (aged ≥18 years old) and adolescents (aged 15–17 years old) completed a written informed consent form. In addition, child participants (aged 7–14 years old) provided written informed assent before being interviewed, in addition to their caregivers’ informed consent. Child participants (aged 5–6 years old) who are lingual provided verbal assent.

## RESULTS

We conducted 30 interviews with 15 children treated for TB and their caregivers. For 13 of the 15 children, caregivers were present at the interview. In total, 13 children completed both visits while 2 only completed their first interview. On average, children were interviewed 41 months (interquartile range [IQR] 28–52) after TB treatment completion. Eight (53%) participants were male. Participants’ median age was 9 years (IQR 7–10); their ages ranged from 5 to 16 years, with 9 children aged 5–10 years and 6 adolescents aged 10–16 years. Five children spoke *isiXhosa*, and the other ten spoke a combination of English and Afrikaans. Two female participants were living with HIV, a 10-year-old girl and a 14-year-old girl. Twelve of the 13 children with microbiologically confirmed TB had drug-susceptible TB, and one multidrug-resistant TB.

### Post-TB physical wellbeing

Although many caregivers and adolescents reported post-TB physical complications, the majority mentioned that they were able to return to ‘normal’ life. Some children and adolescents perceived notable changes in their bodies following their TB episode (see Figure). Children’s caregivers and adolescents reported a range of post-TB physical symptoms, such as tiredness and physical pain (in multiple parts of their bodies), which they attributed to TB (Table 1). A few adolescents expressed concern that they had not been cured of TB due to persistent symptoms, particularly those they associated with their lungs and overall fatigue. Both children and adolescents reported ongoing frustration with their inability to do things they were able to do before their TB disease episode. Some caregivers were concerned that TB had weakened their children’s immune systems, noting that their children had been more susceptible to other infections since completing TB treatment. For example, one caregiver reported that his child often became bedridden from even minor infections following his TB episode. Several caregivers noted that their children were unable to gain weight following the completion of their treatment, suggesting that their children may not have fully recovered. Some children were also diagnosed with HIV or diabetes, which caregivers felt increased vulnerability to illness and complicated recovery. Participants who reported multiple TB episodes indicated that they had not received structured or routine post-TB clinical follow-up after treatment completion. Instead, when they later presented again with symptoms, they were managed as new patients at their local health facilities, undergoing new diagnoses and re-screening for TB without reference to their prior TB history. One child immediately initiated TB preventive therapy after completing his treatment, in line with guidelines given ongoing exposure in the household. The nurses expressed concern that the child was at risk of getting TB disease again, given that he lived with an adult family member who had active TB and was non-adherent to treatment. Only a small proportion of caregivers reported being informed that their child was at elevated risk for developing recurrent TB. Many participants reverted to self-medicating or seeking care from a pharmacist before returning to a local health facility for relief of ongoing symptoms. This was sometimes attributed to a lack of trust or confidence in formal health services, particularly when caregivers felt that previous visits had not led to meaningful care or recognition of their child’s TB history.

**Figure.**
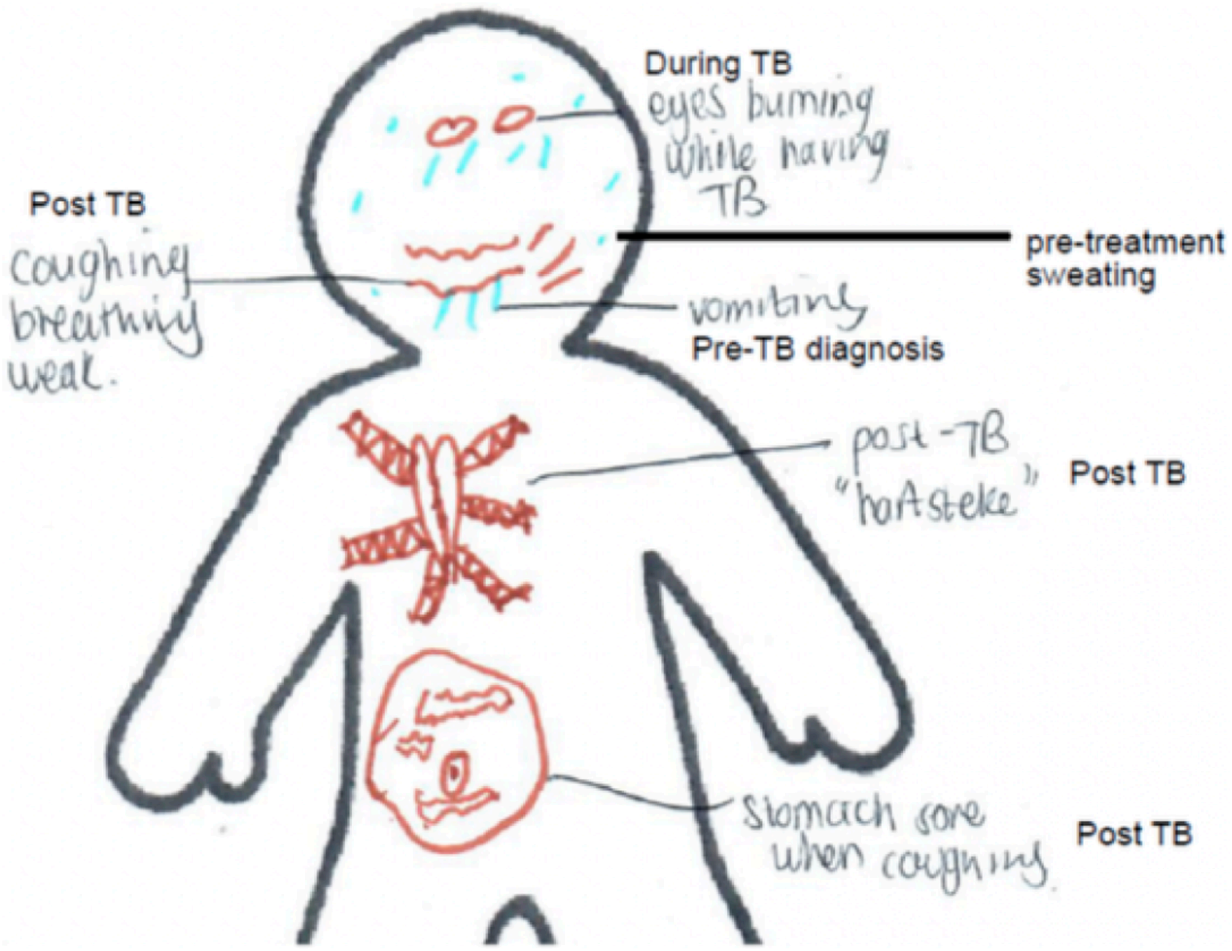
Nine-year-old, boy, body-map activity.

**Table 1.** Illustrative quotes of the physical impact of TB on children, adolescents, and their caregivers’ lives.

Post-TB physical wellbeing	Participants and caregivers’ quotes
Impact on children’s physical activity/abilities	‘I get tired very easily’ (11-year-old girl).
	‘I can’t play netball because I get tired easily’ (14-year-old girl).
	‘When she runs her chest suddenly feels tight, and it closes’ (Caregiver of 10-year-old girl).
	The caregiver of a 9-year-old boy noted that when her son is done playing soccer, he leans forwards on his knees, ‘I can see the difference between him and my younger brother’. Later in the interview the boy said, ‘If I play ball (soccer) then my heart beats, I cough, and I breathe heavily and my stomach hurts. (…) My heart still pains sometimes’ (9-year-old boy).
Ongoing vulnerabilities	When asked if she feels like her children have been affected by TB, the caregiver of a 7-year-old girl noted the following: ‘No I think (…) they are doing very well at the moment. Look (my daughter) gets sick very often, and when she gets sick, she is bedridden, and I believe that is from the TB. (…) (My son) was sick last month and his heart starting paining (shooting pains) again and his eyes were red. Then I said he should lie down for a while, we thought it was a wind at first (…) I don’t really have a trust anymore in the hospital, but he recovered’.
	When we asked the caregiver of a 10-year-old boy if she thinks her son had been cured of TB, she said, ‘In my opinion, I think he’s cured but I think he’s vulnerable to something like that, to get it again or to get any disease again’.
	When we asked the caregiver of a 7-year-old girl if she believes her daughter was cured of TB, she said, ‘No, because I see with my family now. They are healed but then there is a time they pick up the TB again’.
	When asked about TB’s impact on her son’s life, the caregiver of a 9-year-old boy said, ‘I don’t know because they give him that nasal spray every month. It’s two pumps in the evening and two in the mornings. It started after the TB (…) sometimes he gets the headaches now. Then he seems like he can’t do anything because the headache just makes him lie down. (…) if you had TB once you can get it again very quickly, it’s my own stuff, nobody said that to me, and people who maybe have TB and you sit around them’.
	‘His TB apparently left something behind, now he has to be on allergy pills, he gets every month (…) He gets a nasal spray. If it’s so hot then his nose just starts to bleed like that, and his lungs aren’t right yet (…) they give me about 4 months of pills at a time’ (Caregiver of a 9-year-old boy).
Post-TB clinical care	None of the children and caregivers talked about receiving any post-TB clinical care after finishing TB treatment.
	‘I give her (paracetamol) syrup or (tablets) because even if she takes her to the clinic or doctors, they will give her the same thing’ (Caregiver of a 14-year-old girl).
	‘She finished the treatment, and we are not going to the clinic anymore. After she finished the treatment, I took her to Karl Bremer for a check-up. They said she has spots on the liver, but it’s nothing to worry about. We never went again’ (Caregiver of a 14-year-old girl).
	‘I went to the clinic, the nurses said that he had finished his treatment. After he finished his TB treatment, at the clinic they gave him some pills that he took once a day. They said the pills are to protect him from TB. At that time, my uncle who had TB and was still alive, that’s why they gave him these pills to prevent TB. Treatment has helped him a lot, but I don’t like this pill thing because he is growing up on pills, what kind of child is growing up on pills’ (Caregiver of a 7-year-old boy).

### Post-TB psychological wellbeing

Overall, TB and its treatment negatively impacted participants’ psychological wellbeing (Table 2). Major stressors for children included the initial TB diagnosis subsequent hospitalisation, the adverse effects of TB treatment, and ongoing symptoms post-TB treatment completion. Adolescents reported a wide range of emotions: beginning with initial shock and surprise, followed by anger, grief, and hopelessness, eventually leading to resignation or acceptance after some time on treatment. When probed about the possibility of TB recurrence, children and adolescents expressed fear, with some showing signs of distress. Caregivers also reported a negative impact on their own psychological wellbeing after their children’s TB diagnosis. Caregivers’ primary response to their children’s TB journeys was complex, with fear being prominent, but also encompassing expressions of love, care, and resilience: fear of their child dying, contracting TB again, or having their life permanently altered by the disease, alongside a deep commitment to providing care and protection. A caregiver of a 6-year-old girl said, ‘all I fear is that TB affects her lungs, maybe if she wants to do sports in the future’. Caregivers, particularly those who identified as the contact person, reported experiencing immense guilt for transmitting TB to their children. Several caregivers mentioned feeling depressed, with one sharing that she had recently been prescribed sleeping pills to help with her anxiety and would soon start antidepressants and attend counselling.

**Table 2.** Illustrative quotes of the psychological impact of TB on children, adolescents, and their caregivers’ lives.

Post-TB psychological wellbeing	Children, adolescents, and caregivers’ quotes
Emotional response to TB care and treatment	The caregiver of a 9-year-old boy questioned her son about his reaction to his TB treatment: ‘When I picked you up from school when I told you we were going to Tygerberg, then what do you say to me? You said you don’t want to have injections again’. The 9-year-old boy then went on to say, ‘I cried because it hurts (…) I thought I was going to die (…) I was very sick’.
	When we asked a 15-year-old teenage boy how he would feel if he were to have a 2^nd^ TB episode, he said, ‘I would be angry because I don’t want to go through that again’.
Stress and anxiety among caregivers	‘I take sleeping pills because I went into depression and anxiety about 2 weeks ago, and then the doctor gave me sleeping pills because I sleep so little. My brain was overwhelmed, and I was tired. (…) The doctor is going to see if she can put me on anti-depressants. (…) The psychologist wants to work with me herself’ (Caregiver of a 9-year-old boy).
	The caregiver of a 15-year-old teenage girl said she blamed herself for her daughter developing TB, because she was not there to make sure her daughter took her ARVS: ‘If I was present, she would have not gotten TB and since I have been staying with her throughout these years after the diagnosis, she is fine, she did not get TB’.
	When asked how she would feel if her son contracted TB again, the caregiver of a 9-year-old boy said, ‘I’ll feel hurt that he has the same disease again (…) it was hectic to give him and his brother medication, it was a big struggle’.

### TB-stigma’s enduring negative impacts on social wellbeing

Stigma experienced during their disease episode substantially negatively influenced children and adolescents’ social and educational wellbeing, with enduring impacts, particularly among caregivers (Table 3). Participants reported experiencing enacted stigma, as discrimination or pejorative behaviour/language, from friends and community members during treatment. Participants also reported anticipated stigma, expecting mistreatment or discrimination, leading many to isolate themselves from others. Some adolescents chose to isolate themselves and some caregivers chose to isolate their children during TB treatment, and both groups were sometimes encouraged to do so by health workers or family members. Some participants’ family members also enacted stigma, citing fear of TB transmission as justification for isolation. This dynamic led to strained family relationships and significant conflict within the household. The impact and after-effects of TB disease in children were not always fully considered, and caregivers reported feeling misunderstood and not supported by household members. In contrast, other participants noted that family members were loving and supportive, which went well beyond the end of TB treatment, even providing financial assistance to alleviate TB care–related costs.

**Table 3.** Illustrative quotes of the social and educational impact of TB on children, adolescents, and their caregivers’ lives.

Social and educational impact of TB	Children, adolescents, and caregivers’ quotes
Stigma and long-term impact on social relationships	‘She insulted me in the presence of (family members) and said that I have TB and that’s why I can’t go to school, and she even told my friends. After that my friends stopped playing with me and we are no longer friends even now. I was very hurt, and I cried. (…) I only told (a friend) about TB, and I did not tell my other friends. I only told (her) because she also had TB before. Nothing changed about my friends because they didn’t know that I had TB’ (14-year-old girl).
	When we asked the caregiver of a 7-year-old girl about her family’s response to her daughters’ TB diagnosis, she said, ‘I didn’t tell people. I almost want to say I was shy. I asked myself if she was going to infect the other children. (…) For me, it was like this. She was going to infect us all. We didn’t want to touch her. Then I said, no, you shouldn’t (treat her this way). You love her, we (should) give her love’.
	A 14-year-old girl said, ‘I was scared people would look at me in a funny way. I was not comfortable taking the treatment in front of my classmates and other school children because they were going to laugh at me’. The child’s caregiver went on to say, ‘When she did not take the treatment in the morning before school, I would take the treatment to school to make sure she did not miss it. Even the other pills (HIV treatment), she did not want them, we fight a lot about that, those are the things that make us to not get along well’ (Caregiver of a 14-year-old girl).
	The caregiver of a 10-year-old girl said that she did not inform her daughter’s school of her daughter’s TB diagnosis because ‘school children can be cruel. I didn’t want them to know because maybe when they are in conflict, they would insult her with her TB issue’.
Emotional support of the household	The caregiver of a 10-year-old boy described TB as a traumatic event and something which could rob one of one’s humanity, if one isn’t careful about how one approaches it: ‘TB is something that’s very traumatic, it’s something that’s very sensitive and there’s so much around TB that it takes so much of one’s humanity. TB is very big to me it’s not something that should be taken very small or small because it takes a lot of one’s humanity I can see sometimes people’s lives changed after having TB, it can be good or negative because I, look as I have seen by now people who had TB who used to be very weak and now they are stronger and then you get people who were very strong and who are now much weaker, TB is not something to take lightly’.
	‘They know about TB, and they know if they are on treatment they will get better when their treatment is finished (…). But they don’t understand about the after-effects, or the traumatic or the depression you go through. They have no idea how dangerous it is’ (Caregiver of a 9-year-old boy).
	When asked to reflect on the support she received from her family and neighbours, the caregiver of a 12-year-old girl said, ‘No, I don’t really complain. They supported us well during that time. They never behaved differently/strangely towards us’.
	‘(since TB) he takes care of us, makes sure we get everything we want, when every time we are sick, he makes sure it’s going to be okay, my grandfather takes us to the hospital’ (9-year-old boy).
	The caregiver of a 7-year-old girl said that she sometimes fights with her partner and other household members due to financial strain: ‘Oh we fight sometimes, over money mostly. You know, I’m just praying the Lord will help us’.

For some participants, the unpredictability of sharing their diagnosis with others led them to keep their TB diagnosis a secret. Even after completing treatment, a few participants remained cautious about disclosing that they had had TB, preferring that others remain unaware of it. This was evident not only in the way participants spoke about themselves but also in the changes to household membership. In some cases, children experienced temporary, short-term moving of homes, immediately following diagnosis, to avoid the risk of future TB disease – sometimes even leaving their caregivers’ homes – revealing underlying perceptions of risk and fear, as well as concerns about maintaining a healthy environment for their children. Caregivers highlighted several challenges linked to their home environments that influenced their experience of caring for children with TB.

### Interruptions to schooling and the school-associated peer relationships/social wellbeing

Multiple participants reported the negative impacts TB had on schooling. Some reported being hospitalised for several weeks at the start of their TB treatment, while others mentioned ‘losing’ a year of schooling and having to repeat a grade due to extensive absences (Table 4). In some cases, post-TB physical complications negatively affected children’s school attendance. Not graduating at the expected age could delay income generation and negatively impact children’s and adolescents’ future financial opportunities. Additionally, being away from school for long periods (up to 3 months) negatively affected their social lives, with many citing the loss of friendships.

**Table 4.** Illustrative quotes of the impact of TB on children and adolescents’ schooling and school-associated peer relationships/social wellbeing.

Social and educational impact of TB	Children, adolescents, and caregivers’ quotes
Long-term impact on schooling	‘When it’s very cold, I didn’t allow her to attend school because she would get sick very fast, I even told her class teacher’ (Caregiver of a 10-year-old girl).
	‘He was still young, when he had TB, he had not started TB school yet and it did not affect his future. If maybe he had already started school, maybe we would have seen the difference’.
	‘The doctors didn’t tell me about the side effects. After the first time and the second time he had (his treatment), he was very tired, and he had trouble gaining (weight). He is (now) full of energy, and he runs around, but he gets tired soon. All I fear is that (TB) affects his lungs, maybe if he wants to do sports in the future’ (Caregiver of a 9-year-old boy).
Short- and long-term economic burden of TB	‘Sometimes I would pray that (the child support) social grants be paid closer to clinic visits’ (Caregiver of a 6-year-old girl).
	‘Sometimes I don’t even have money to go to the clinic (transport), to collect her pills, I have to borrow from neighbours and friends’ (Caregiver of a 10-year-old girl).
	‘At that time, I was unemployed, during COVID, dependent on a grant, and had no money to buy cool drink’ (Caregiver of a 10-year-old girl).

## DISCUSSION

Our study is among the first to qualitatively describe children, adolescents, and their caregivers’ post-TB experiences. Our findings highlight how the physical, psychological, and social effects of TB extend beyond TB treatment completion. Children and adolescents reported enduring challenges, such as impaired physical function, emotional distress, and social isolation/exclusion. Children and adolescents reported disruptions to education, while caregivers reported economic hardship, even after TB treatment completion. Our findings align with other empirical studies of young child and adolescent TB survivors that show long-term physical impairment.^7–9,15^ Several caregivers expressed concerns that TB treatment had weakened their child’s immune system, making them more vulnerable to other infections. Although many participants, including children, their caregivers, and adolescents, cited TB as the cause of ongoing ill health and vulnerability, it was difficult to discern post-TB symptoms from general health issues, including pre-existing conditions such as asthma or allergies or other common childhood illnesses.

Nearly all the children and adolescents reported distress related to their TB diagnosis and treatment journey. Children and adolescents’ ongoing psychological challenges, such as fear and anxiety, appeared to be linked to the treatment processes and isolation because of stigmatisation – as well as the possibility of future new TB episodes. Children and adolescents’ social lives were negatively impacted by TB, both during and after treatment completion. Some reported that stigma led to disrupted friendships and strained family relationships, while others said that they were provided ample support and care throughout their TB journeys. Children and adolescents reported interruptions in their schooling even though current guidelines do not recommend prolonged isolation.^1^ Our findings are similar to those of Wademan et al.^26^ who reported the profound impacts that TB has on children and adolescents’ social wellbeing. Our findings demonstrate the long-lasting duration on these impacts and suggest that further research in this area is high priority.

Caregivers interviewed about their children’s TB conditions frequently expressed feelings of guilt and exhibited signs of mental distress. These findings align with previous research conducted in Cape Town, South Africa, where caregivers of children receiving TB preventive therapy reported experiencing psychosocial challenges, including feelings of guilt and shame for having exposed their child to household contacts with TB, alongside the withdrawal of social support from family members.^29^ Our findings emphasise the enduring negative effects of TB on the lives and psychosocial wellbeing of children and adolescents. Social support and stigma played significant roles in their experiences.^27^ A multi-stakeholder study recently completed in The Gambia, that included adolescents, established that adolescents experience long-term physical challenges and ongoing disruptions to their social lives post-TB treatment completion.^30^ Unlike this multi-stakeholder study, children and adolescents in our study acknowledged experiencing stigma during treatment and a few, particularly those who had TB in late childhood or adolescence, suggested that this stigma may persist after treatment completion. This warrants further exploration. This differs from previous research among adults with TB, where participants’ accounts indicated that TB had ‘stained’ them.^31^ The differing levels of concern about ongoing stigma among children, adolescents, and adults could be attributed to various factors, including data collection methods, cultural contexts, and different degrees of social participation and perceived risk of infectiousness. Additionally, children and adolescents may not fully recognise or express long-term stigma due to psychosocial development factors, as they are at a different stage of understanding and processing social experiences compared with adults. It also may be that children and adolescents are perceived as innocent victims of TB, whereas adults are seen as responsible for their health and thus blamed for contracting TB, thereby exempting children and adolescents from TB-related stigma.^21,32–34^

Strengths of the study include that data were collected through multiple interactions with children, adolescents, and caregivers, offering a holistic perspective of their experiences post-TB treatment. The qualitative nature of the study allowed for an in-depth exploration of experiences, yielding rich insights into their physical, mental, and social health post-TB. Purposive sampling enabled the recruitment of children and adolescents who had TB at various ages, allowing us to capture a diverse range of post-TB experiences. Several limitations should be noted. The sample size, though valuable for qualitative insights, may not allow for broader generalisations to larger populations. Some children and adolescents had TB more than 5 years before participating in this study, which may introduce recall bias regarding their TB treatment experiences. A few children were also very young at the time of the interview, which means that some questions may have been difficult to understand, and caregivers had to serve as proxies. However, the use of participatory research methods, such as body-map activity, may have helped address this challenge by offering more engaging ways for children to connect with, to understand the questions, and to express themselves, even when verbal communication might have been limited. Future research needs to leverage even more child-friendly research mechanisms to better capture post-TB experiences of young survivors.

A key implication of our findings is the need to better understand the challenges faced by children and adolescents after TB treatment, especially the long-term consequences for their physical health, psychosocial wellbeing, and education. The findings provide impetus for further studies in additional settings and with longitudinal designs to expand qualitative and descriptive research on post-TB experiences within paediatric populations across diverse settings, to inform tailored programmatic interventions. This is crucial to better understand how the impact of TB may vary based on contextual factors.

## Supplementary Material


