# Supplementary material for: Post-TB treatment completion experiences of children, adolescents, and caregivers from Cape Town
Source: IJTLD Open. 2025 Nov 12;2(11):662–70. doi: 10.5588/ijtldopen.25.0117 (PMC12617085; doi:10.5588/ijtldopen.25.0117)
Supplement: Supplementary file 1 [file ijtldopen25-0117_supplementarydata1.pdf]

CAPEs Discussion Guide

Purpose: The quality-of-life impact of post-tuberculosis lung disease in children and adolescents is an in-depth evaluation of the impact of tuberculosis (TB) on children and adolescents' lives. The aim of this discussion is to understand the child and caregivers' experiences of TB disease, treatment and long-terms affects. The interview guide is to facilitate a discussion about key topic areas but should be used flexibly with each participant over the course of the interactions with them. It is to be used alongside the ARF and RIDD.

Objectives:

1. To describe children's and adolescents' experiences post-tuberculosis treatment and/cure.
2. To describe how children's and adolescents' household contexts impact their experiences post-tuberculosis.
3. To describe children's and adolescents' perceptions of tuberculosis's impact on their quality of life including, physical, psychological, social, educational and financial aspects of their lives.
4. To describe children's and adolescents' experiences of and perceptions of post-tuberculosis rehabilitation.

Form of data recording: (1) Audio-recording of all talk from "Preamble" to "Closing". (2) Notes of key points per topic area handwritten by the facilitator into a printed copy of this document. (3) Still photographs of relevant images.

Expected time needed per use: 45-60 mins

Preamble (**to be read by facilitator**): Today is the (**insert date [day xxth Xxx xxxx**]) and it is (**insert time XX:XX**). This is a discussion with (**insert participant**) who is enrolled in the Omoya study. Thank you for your time. These discussions are to understand the impact that tuberculosis has on the lives of children and adolescents.

During this interview, we would like to find out more about who you are, where you live, and your broader TB experience. As discussed during the consenting process, we are audio recording this discussion. Please could you speak loudly and clearly, to ensure that we can hear you on the recording? I will be taking some notes to help facilitate this discussion. These notes are to help me keep track of our conversation and remind me to ask more details about your experiences. Do you have any questions before we begin?

Visit 1 – Getting to know the participant and their household

Topic area 1 – Kinship mapping

To draw a picture that represents (1) the participants' "family" (biological and non-biological), (2) the types of relationships between each member of that "family", and (3) cohabitation/co-residence of people ("family" and non-family) with the participant.

Suggested probes:

- Are all household members and family members aware that the child is on TB preventative therapy treatment and that the child is enrolled in S35?
- Have there been newer members in household because the child is on TPT? *(are there people assisting the caregiver? Is the caregiver present, the only caregiver to the child?)*

- How has your child's inclusion in the study, impacted the relationships within the home? *(are there people who have since moved out? Are there people whose roles have changed?)*

Topic area 2 – Timeline – history of TB in the family

To understand your family's history of TB and other diseases over time.

Suggested probes:

- Could you please tell us more about your child's treatment journey?
- When did you first learn about TPT?
- What did they tell you about TPT at the clinic?
- Did your child receive TPT from a local clinic before they started on TB-CHAMP? Tell me about that.
- If so, how long was your child on that treatment? Tell me more about the treatment. (Was it easy to prepare and administer? Did your child like the treatment? What did it taste like? What are some of the challenges you experienced with the TPT you received from the clinic?)

Topic area 3 – Social network activity

To create a platform to discuss and understand the participants' social network and the relationships the participant engages in, by visually representing kinship, friendship, romantic, communal and acquaintance networks of the participant.

Suggested probes:

- Could you please tell us more about your social network (friends and community):
- What is your community like? (Do people spend a lot of time together?)
- How often do your friends come over? Where does your child spend a lot of time? Who does your child spend a lot of time with?
- Did anything change with your friends, when [index case] found out they had MDR-TB?
- Did anything change with your friends, or your child's friends, when your child started taking the study drug?

Visit 2 – Experiences and quality of life after TB

Topic area 4 – Body mapping

To draw a picture that represents (1) the participants' "body"; (2) to explore how the participant perceives their body; 3) to understand how the participants experience of the 3HP regimen; and 4) to explore the participant's emotional-physical-social experience because of being on TB preventative therapy treatment.

Suggested probes:

- Have you / Has your child experienced any physical side effects to the treatment? *(reports of vomiting and nausea? Weight loss? Loss of appetite? Hyperpigmentation?)*
- Do you / Does your child prefer the taste of the treatment? *(does it taste bitter or sweet? Do you like the taste? What would you have preferred in tastes like?)*
- How has the emotional wellbeing of the child been affected since enrolment in the study? *(have they been seemingly sad, fatigue, tired etc. have there been changes in mental state? Has the child expressed any emotions towards being enrolled in the study and being on TPT treatment?)*
- Do you think being on the Study35 TPT treatment has had any impact on your child's view of themselves? *(has your child expressed any concerns about other people's thoughts on them, being on treatment?)*
- Has your child ever been treated differently because of being on TPT treatment? *(who, besides family members, knows about your child being on TPT? Those who know, how has their behaviour been since knowing?)*

Topic area 5 – Health beliefs / conceptualisation of health activity

To identify the ways in which participants conceptualise health by (1) exploring the ways in which healthy/sick bodies are described, (2) exploring key concepts related to health and illness and (3) to describe how these concepts relate to each other.

Topic area 6 – Quality of life: card-sorting activity

To identify the ways in which participants think Quality of Life (QoL) is by representing (1) the most important domains of quality of life for the participant and (2) the most important ideas/aspects of QoL for each domain.

Topic area 7 – Parameters of ambitions activity

To understand the parameters of ambition of participants which take the form of hopes, dreams, ambitions, and fears. By discussing these parameters, we may illicit the motivations for general health seeking behaviour such as pursuing/not pursuing health care, adherence, and treatment acceptability. The participants response to this activity aids in understanding their experiences of illness, adherence, disclosure, and other health related experiences and assists in the progress of treatment programmes.
